# A 24-month retrospective update: follow-up hospitalization charges and readmissions in US lumbar fusion surgeries using a cellular bone allograft (CBA) versus recombinant human bone morphogenetic protein-2 (rhBMP-2)

**DOI:** 10.1186/s13018-021-02829-0

**Published:** 2021-11-18

**Authors:** Bradley Wetzell, Julie B. McLean, Kimberly Dorsch, Mark A. Moore

**Affiliations:** 1 Global Scientific Affairs and Clinical Engagement, LifeNet Health®, 1864 Concert Drive, Virginia Beach, VA 23453 USA; 2 Global Clinical Affairs, LifeNet Health®, Virginia Beach, VA USA

**Keywords:** Cellular bone allograft, CBA, Economics, Lumbar fusion, Infuse, Recombinant human bone morphogenetic protein-2, rhBMP-2, ViviGen

## Abstract

**Background:**

The objectives of this study were to build upon previously-reported 12-month findings by retrospectively comparing 24-month follow-up hospitalization charges and potentially-relevant readmissions in US lumbar fusion surgeries that employed either recombinant human bone morphogenetic protein-2 (rhBMP-2) or a cellular bone allograft comprised of viable lineage-committed bone cells (V-CBA) via a nationwide healthcare system database.

**Methods:**

A total of 16,172 patients underwent lumbar fusion surgery using V-CBA or rhBMP-2 in the original study, of whom 3,792 patients (23.4%) were identified in the current study with all-cause readmissions during the 24-month follow-up period. Confounding baseline patient, procedure, and hospital characteristics found in the original study were used to adjust multivariate regression models comparing differences in 24-month follow-up hospitalization charges (in 2020 US dollars) and lengths of stay (LOS; in days) between the groups. Differences in potentially-relevant follow-up readmissions were also compared, and all analyses were repeated in the subset of patients who only received treatment at a single level of the spine.

**Results:**

The adjusted cumulative mean 24-month follow-up hospitalization charges in the full cohort were significantly lower in the V-CBA group ($99,087) versus the rhBMP-2 group ($124,389; *P* < 0.0001), and this pattern remained in the single-level cohort (V-CBA = $104,906 vs rhBMP-2 = $125,311; *P* = 0.0006). There were no differences between groups in adjusted cumulative mean LOS in either cohort. Differences in the rates of follow-up readmissions aligned with baseline comorbidities originally reported for the initial procedure. Subsequent lumbar fusion rates were significantly lower for V-CBA patients in the full cohort (10.12% vs 12.00%; *P* = 0.0002) and similar between groups in the single-level cohort, in spite of V-CBA patients having significantly higher rates of baseline comorbidities that could negatively impact clinical outcomes, including bony fusion.

**Conclusions:**

The results of this study suggest that use of V-CBA for lumbar fusion surgeries performed in the US is associated with substantially lower 24-month follow-up hospitalization charges versus rhBMP-2, with both exhibiting similar rates of subsequent lumbar fusion procedures and potentially-relevant readmissions.

## Introduction

Lumbar spine fusion is widely used to treat back pain when more conservative treatments have failed [1, 2]. While autologous bone grafts have been historically preferred due to their presumed ability to provide all three components of bone healing (ie, osteoconductivity, osteoinductivity, and osteogenicity) [3], their supply is limited and their quality is potentially constrained by patient comorbidities and other lifestyle risk factors [4]. Further, the additional surgical procedure increases operative time, blood loss, risk of infection, and postoperative pain [4]. As an alternative, recombinant human bone morphogenetic protein-2 with a bovine collagen sponge scaffold (rhBMP-2; marketed as Infuse® by Medtronic Inc., Memphis TN), has been widely utilized with demonstrated osteoinductive efficacy in spinal fusion, despite US FDA approval for the spine being limited to single-level lumbar fusions within approved interbody cages and multiple reports of serious complications [5–7]. However, rhBMP-2 remains relatively expensive [8–10] and reduction of economic burden has become an increasingly high priority in modern healthcare systems [5].

Cellular bone allografts (CBAs) are another alternative that are theoretically more similar to autograft in that they are also presumed to be osteoinductive, osteoconductive, and osteogenic [11, 12]. While most CBAs rely on viable cryopreserved mesenchymal stem cells for their osteogenic component, a more recent advanced CBA (V-CBA; marketed as ViviGen® by LifeNet Health®, Virginia Beach VA) is comprised of lineage-committed bone-forming cells, which may be more conducive to effect bone fusion [13–15]. V-CBA can be used for homologous repair of any bone defect throughout the body [16] and reported clinical outcomes of spinal fusions using V-CBA have thus far been positive [10, 12].

Previously, we reported [10] that use of V-CBA was associated with $51,130 less in mean hospital charges for initial lumbar fusion procedures versus rhBMP-2, and $22,091 less in mean 12-month follow-up hospitalization charges. Yet, these 12-month data showed that patients receiving either graft exhibited similar rates of subsequent lumbar fusion procedures and potentially-relevant hospital readmissions [10]. The primary objective of this study was to build upon these findings by extending the comparison to 24-months of follow-up hospitalization charges and resource utilization. The secondary objective was to assess the 24-month incidence of potentially-relevant follow-up readmissions.

## Materials and methods

### Study design, data source, and patient selection

This was a retrospective cohort study based on previously-published work [10] and using data from the Premier Healthcare Database (PHD; Premier Healthcare Solutions, Inc.; Charlotte NC). The PHD is a US hospital-based, service-level, all-payer database with a geographically diverse, nationwide footprint [17]. At the time of the original study, the PHD contained standard discharge data (including patient demographics, disease status, and date-encoded billed services) for approximately 208 million unique patients from over 1,000 hospitals. Within-system activities for a given patient were tracked across visits using unique patient and encounter identification codes, which did not contain personally-identifiable information. Data from the PHD are thus considered deidentified in accordance with the HIPAA Privacy Rule described in Title 45 of the US Code of Federal Regulations (CFR) Part 164.506(d)(2)(ii)(B) and are exempt from Institutional Review Board oversight, as provided in 45 CFR 46.101(b)(4) [2, 17].

Lumbar fusion procedures from the original study occurred from 01 October 2015 through 30 September 2018, and related analyses of those and 12-month follow-up readmissions (through 30 September 2019) were reported previously [10]. For the present study, an updated extract of PHD data from 01 October 2015 through 30 September 2020 was acquired to permit an extended analysis of follow-up patient activities over a 24-month period.

Per PHD standard procedure, data for patients meeting any of the following criteria were excluded from both extracts: patients not at least 18 years of age at the time of the initial procedure, patients from hospitals that did not continuously report to the PHD throughout the follow-up period, and patients who died during the initial admission. Given that at least one of these exclusion criteria could potentially introduce variation between extracts over time, only data from the most recent extract were used for the present analyses. Thus, although predicated on the dataset of patients and initial surgeries identified in the original study [10], the present 24-month follow-up readmissions dataset should be considered otherwise independent of the originally-reported 12-month follow-up dataset.

Specifically, patients who underwent initial lumbar fusion procedures using V-CBA or rhBMP-2 in the original study were identified in the new data extract via their unique patient identification codes. The initial surgeries from the original study were then located using the unique encounter identification codes and the recorded dates of service were used to designate inpatient encounters during the subsequent 24-month period for each patient as all-cause follow-up readmissions. Importantly, these data did not include patients who may have received follow-up treatment outside of the Premier Healthcare System.

### Study variables and statistical methods

Baseline patient, procedure, and hospital characteristics that were assessed and reported in the original study [10] included age, sex, race, ethnicity, Charlson comorbidities, health insurance status, initial admission type, initial admission source, initial discharge status, cage insertion, multiple levels treated, hospital size, hospital teaching status, hospital population served, and hospital region. Differences in these characteristics that were originally identified between the V-CBA and rhBMP-2 groups at the time of the initial surgery [10; Table 1] determined which confounding factors were treated as covariates in the present primary analyses.

**Table 1:** 24-month Follow-up Hospital Readmission Lengths of Stay

No. days	Full cohort			Single-level cohort		
	Group		*P* value	Group		*P* value
	V-CBA (n = 1,522)	rhBMP-2 (n = 2,270)		V-CBA (n = 1,076)	rhBMP-2 (n = 1,531)	
Unadjusted cumulative mean^a^	8.16	7.15	0.0252*	8.16	7.07	0.1453
(SD)	(12.61)	(9.07)		(13.78)	(9.04)	
Adjusted cumulative mean^b^	7.71	7.45	0.4863	7.78	7.34	0.3595
(95% CI)	(7.15, 8.27)	(6.99, 7.15)		(7.08, 8.48)	(6.76, 7.91)	

Readmission data did not include patients who may have received follow-up treatment outside of the Premier Healthcare System

^a^Wilcoxon rank-sum test

^b^Multivariate regression models were adjusted with the following confounding factors identified in the original study: race, ethnicity, Charlson comorbidity index, health insurance status, initial admission type, initial admission source, initial discharge status, cage insertion, multiple levels treated (full cohort only), hospital size, hospital teaching status, hospital population served, and hospital region

*Statistically significant

For the primary objective, cumulative hospitalization charges (in 2020 US dollars) and reported lengths of stay (LOS; in days) were calculated for each patient during the 24-month follow-up period and unadjusted means and standard deviations (SDs) were reported for each group. Differences in hospitalization charges and LOS between the V-CBA and rhBMP-2 groups were assessed using multivariate regression models adjusted with the originally-reported confounding baseline factors as covariates, and the resulting adjusted means and 95% confidence intervals (CIs) were presented.

For the secondary objective, potentially-relevant follow-up readmissions were assessed as the following procedural and diagnostic variables, which were defined by ICD-10 codes as reported previously [10; Page 7]: subsequent lumbar fusion procedures, cardiac complications, deep vein thrombosis, hematoma, nervous system complications, pneumonia, pulmonary embolism, sepsis, surgical-site infection, and urinary tract infection. The incidence of each complication during the 24-month follow-up period was presented as number and percentage of patients within each group, and comparisons between the V-CBA and rhBMP-2 groups were conducted using Fisher’s exact tests.

Similar to the ad hoc analyses described in the original study [10], and to address the potential for greater variation in charges and outcomes for multiple-level surgeries, all primary and secondary analyses were repeated for the subset of patients whose initial lumbar surgery involved only a single level of the spine. This single-level cohort was originally isolated by removing patients from the full cohort with initial surgeries designated with ICD-10 procedural codes containing 0SG1% (fusion of 2 or more lumbar vertebral joints). As with the full-cohort analyses, differences between groups in baseline characteristics that were identified in the single-level cohort of the original study [10; Table 1] determined which confounding factors were treated as covariates in the present primary analyses within the single-level cohort.

Statistical analyses were performed using STATA software, Version 16, (StataCorp Inc, College Station TX). Statistical assumptions were verified as appropriate for each statistical test and significance was assessed at the 0.05 alpha level.

## Results

### Patients

**Fig. 1:**
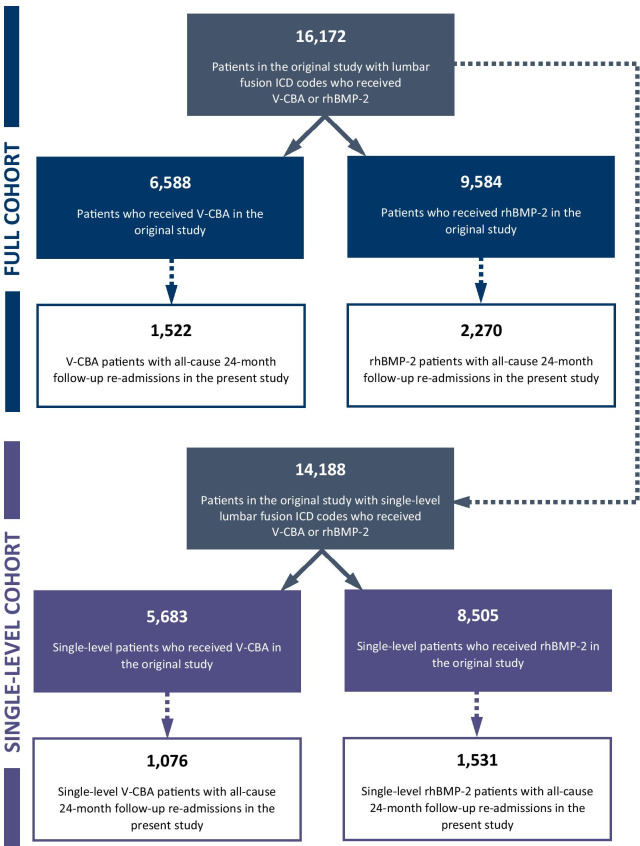
Data-selection flow chart for patients from the original study (full and single-level cohorts) with all-cause 24-month follow-up readmission data in the present study. Data did not include patients who may have received follow-up treatment outside of the Premier Healthcare System

The data-selection flow-chart for this study is presented in Fig. 1. As reported previously [10], there were 16,172 patients in the original study who underwent lumbar fusion surgeries involving V-CBA or rhBMP-2 within the specified date range, of whom 6,588 patients received V-CBA and 9,584 patients received rhBMP-2. Of these, there were 1,522 patients (23.1%) from the V-CBA group and 2,270 patients (23.7%) from the rhBMP-2 group identified in the current study with all-cause readmissions during the 24-month follow-up period. For the single-level cohort, there were 14,188 patients in the original study, of whom 5,683 patients received V-CBA and 8,505 patients received rhBMP-2 [10]. Of these, there were 1,076 single-level patients (18.9%) from the V-CBA group and 1,531 single-level patients (18.0%) from the rhBMP-2 group identified in the current study with all-cause readmissions during the 24-month follow-up period.

### Baseline patient, procedure, and hospital characteristics

The distributions and statistical comparisons of baseline patient, procedure, and hospital characteristics were described previously [10; Table 1]. Briefly, the mean patient age (SD) for each group in the full cohort was V-CBA = 60.86 (13.13) years and rhBMP-2 = 60.74 (13.46) years, and the majority of patients in each group were female, white, and non-Hispanic [10]. Of note, the original mean Charlson Comorbidity Index (CCI) score (SD) at the initial procedure in the full cohort was significantly higher in the V-CBA group (0.92 [1.39]) than in the rhBMP-2 group (0.78 [1.20]; *P* < 0.0001, Wilcoxon rank-sum test), including significantly-higher incidences in the V-CBA group for the following specific comorbidities: any malignancy, cerebrovascular disease, chronic obstructive pulmonary disorder, diabetes with and without complications, hemi- or paraplegia, metastatic solid tumor, myocardial infarction, peptic ulcer disease, and peripheral vascular disease [10]. The distributions and statistical comparisons of baseline characteristics originally reported in the single-level cohort were similar [10], and CCI scores (SD) remained significantly higher in the V-CBA group (0.90 [1.38] vs rhBMP-2 = 0.78 [1.20]; *P* < 0.0001, Wilcoxon rank-sum test) with an additional significantly-higher incidence of comorbid congestive heart failure in the single-level V-CBA group [10].

Additionally, statistical comparisons in both cohorts of the original study identified the following confounding baseline characteristics [10; Table 1], which were treated as covariates in the present multivariate regression models for 24-month follow-up hospitalization charges and LOS: race, ethnicity, Charlson comorbidity index, health insurance status, initial admission type, initial admission source, initial discharge status, cage insertion, multiple levels treated (full cohort only), hospital size, hospital teaching status, hospital population served, and hospital region.

### Hospitalization charges and lengths of stay

The unadjusted mean cumulative hospitalization charges (SD) for the 24-month follow-up period in the full cohort were $102,928 ($115,576) for the V-CBA group and $121,813 ($138,610) for the rhBMP-2 group (*P* = 0.0009, Wilcoxon rank-sum test). In the single-level cohort, unadjusted mean cumulative hospitalization charges (SD) for the 24-month follow-up period were $106,905 ($123,243) for the V-CBA group and $123,906 ($143,326) for the rhBMP-2 group (*P* = 0.0408).

**Fig. 2:**
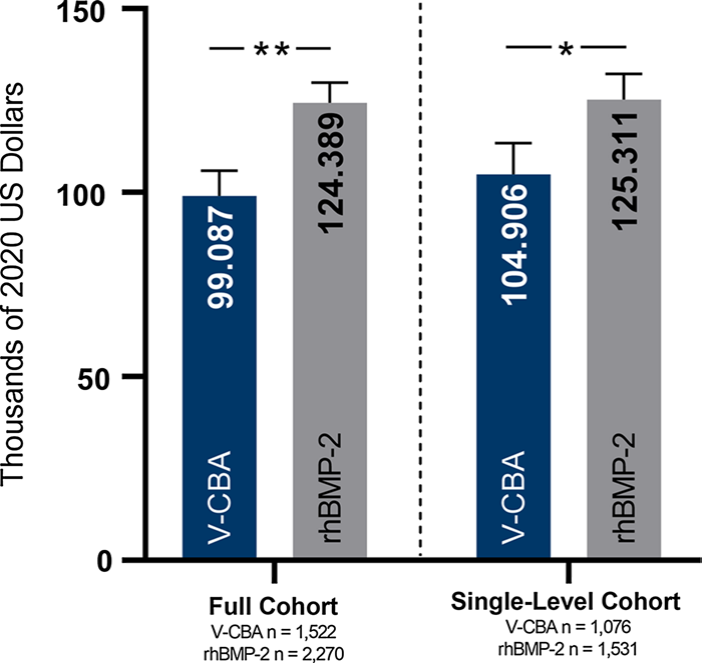
Adjusted cumulative mean 24-month follow-up readmission hospital charges (95% CIs) were significantly lower with V-CBA versus rhBMP-2 in the full and single-level cohorts. **P < 0.0001; *P = 0.0006. Multivariate regression models were adjusted with the following confounding factors identified in the original study: race, ethnicity, Charlson comorbidity index, health insurance status, initial admission type, initial admission source, initial discharge status, cage insertion, multiple levels treated (full cohort only), hospital size, hospital teaching status, hospital population served, and hospital region. Readmission data did not include patients who may have received follow-up treatment outside of the Premier Healthcare System

The adjusted cumulative mean hospitalization charges (95% CIs) for the 24-month follow-up period in the full and single-level cohorts are presented in Fig. 2. After adjusting for confounding factors, the cumulative mean 24-month follow-up hospitalization charges (95% CIs) in the full cohort were significantly lower in the V-CBA group ($99,087 [$92,195–105,979]) versus the rhBMP-2 group ($124,389 [$118,863–129,914]; *P* < 0.0001). This pattern remained in the single-level cohort, where the adjusted cumulative mean 24-month follow-up hospitalization charges (95% CIs) were significantly lower in the V-CBA group ($104,906 [$96,380–113,431]) versus the rhBMP-2 group ($125,311 [$118,289–132,334]; *P* = 0.0006).

The 24-month follow-up LOS for the full and single-level cohorts are summarized in Table 1. In the full cohort, the unadjusted cumulative mean LOS (SD) were 8.16 (12.61) days for the V-CBA group (range 0–263 days) and 7.15 (9.07) days for the rhBMP-2 group (range 1–152 days; *P* = 0.0252, Wilcoxon rank-sum test). Unadjusted cumulative mean LOS (SD) in the single-level cohort were 8.16 (13.78) days for the V-CBA group (range 0–226 days) and 7.07 (9.04) days for the rhBMP-2 group (range 1–152 days; not significant [ns]). After adjusting for confounding factors, no differences were observed for the cumulative mean 24-month follow-up LOS (95% CIs) between the V-CBA (7.71 [7.15–8.27] days) and rhBMP-2 groups (7.45 [6.99–7.15] days; ns) in the full cohort, or in the single-level cohort (V-CBA = 7.78 [7.08–8.48] days; rhBMP-2 = 7.34 [6.76–7.91] days; ns).

### Potentially-relevant Follow-up Readmissions

The distributions and statistical comparisons of potentially-relevant 24-month follow-up readmissions in both cohorts are presented in Table 2. In the full cohort, the 24-month incidence of subsequent lumbar fusion procedures was significantly lower in the V-CBA group (10.12%) versus the rhBMP-2 group (12.00%; *P* = 0.0002, Fisher’s exact test), while the incidences were significantly higher in the V-CBA group for cardiac complications (V-CBA = 0.82% vs rhBMP-2 = 0.37%; *P* = 0.0002) and pneumonia (V-CBA = 1.44% vs rhBMP-2 = 0.99%; *P* = 0.0114). However, these latter two differences corresponded with those reported in the original study for individual comorbidities in the V-CBA group at the initial procedure [10; Table 1], which could not be controlled in this binary analysis. In the single-level cohort, there were no differences between groups in the incidence of subsequent lumbar fusion procedures (V-CBA = 8.53% vs rhBMP-2 = 8.01%; ns), and the significantly higher incidences in the V-CBA group for cardiac complications (V-CBA = 0.63% vs rhBMP-2 = 0.25%; *P* = 0.0022) and pneumonia (V-CBA = 1.16% vs rhBMP-2 = 0.66%; *P* = 0.0113) remained. As in the full cohort, these differences corresponded with those reported in the single-level cohort of the original study for individual comorbidities in the V-CBA group at the initial procedure [10; Table 1]. In both cohorts, the incidences of all other potentially-relevant readmissions, including deep vein thrombosis, hematoma, nervous system complications, pulmonary embolism, sepsis, surgical-site infection, and urinary tract infections were similar between groups.

**Table 2:** Incidence of potentially-relevant 24-month follow-up readmissions

Readmissions, n (%)^a^	Full cohort			Single-level cohort		
	Group		*P* value	Group		*P* value
	V-CBA (n = 6,588)	rhBMP-2 (n = 9,584)		V-CBA (n = 5,683)	rhBMP-2 (n = 8,505)	
Patients with all-cause 24-month follow-up readmissions^b^	1,522 (23.1)	2,270 (23.7)	0.3954	1,076 (18.9)	1,531 (18.0)	0.1635
Re-admitted patients with potentially-relevant procedures/diagnoses^c^						
Subsequent lumbar fusion procedures	667 (10.12)	1150 (12.00)	0.0002*	485 (8.53)	768 (8.01)	0.3191
Cardiac complications	54 (0.82)	35 (0.37)	0.0002*	36 (0.63)	24 (0.25)	0.0022*
Deep vein thrombosis	6 (0.09)	4 (0.04)	0.3344	4 (0.07)	4 (0.04)	> 0.9999
Hematoma	23 (0.35)	31 (0.32)	0.7831	16 (0.28)	22 (0.23)	0.8687
Nervous system complications	19 (0.29)	15 (0.16)	0.0813	16 (0.28)	12 (0.13)	0.0813
Pneumonia	95 (1.44)	95 (0.99)	0.0114*	66 (1.16)	63 (0.66)	0.0113*
Pulmonary embolism	31 (0.47)	41 (0.43)	0.7191	25 (0.44)	32 (0.33)	0.5891
Sepsis	2 (0.03)	5 (0.05)	0.7081	1 (0.02)	3 (0.03)	0.6540
Surgical-site infection	20 (0.30)	23 (0.24)	0.4417	11 (0.19)	14 (0.15)	0.6879
Urinary tract infections	149 (2.26)	185 (1.93)	0.1594	91 (1.60)	127 (1.33)	0.6261

^a^All percentages were based on the total number of patients within each cohort who received V-CBA or rhBMP-2 during the initial procedure

^b^Patients with more than one readmission were counted only once. Did not include patients who may have received follow-up treatment outside of the Premier Healthcare System

^c^Repeats of the same procedure/diagnosis were counted only once. Did not include patients who may have received follow-up treatment outside of the Premier Healthcare System

^*^Statistically significant, Fisher’s exact test

## Discussion

The primary objective of this study was to build upon previously-reported 12-month findings [10] by comparing 24-month follow-up hospitalization charges and resource utilization in US lumbar fusion surgeries using rhBMP-2 versus V-CBA. The secondary objective was to assess the 24-month incidence of potentially-relevant follow-up readmissions. Combined, these were the first known studies to provide direct comparisons of these grafts in lumbar fusion surgeries using a nationwide all-payer healthcare database to access a large, geographically-diverse patient population via real-world economic and clinical data.

In the present study, adjusted cumulative mean 24-month follow-up hospitalization charges were significantly lower in the V-CBA group versus the rhBMP-2 group for both cohorts, with a difference of $25,302 in the full cohort and $20,405 in the single-level cohort (Fig. 2). These disparities were not explained by resource utilization in the form of LOS in this study, for which no differences were found in either cohort. Yet, they remain consistent with those of the original study [10], which found significant reductions in hospitalization charges for initial lumbar fusion surgeries using V-CBA ($51,130 lower with V-CBA vs rhBMP-2) and in cumulative 12-month follow-up hospitalization charges ($22,091 lower with V-CBA vs rhBMP-2). While a recent report by Dietz and colleagues [18] found no difference in average cumulative third-party payments at 24 months following (and including) spinal deformity surgeries using rhBMP-2 ($141,664) vs no rhBMP-2 ($144,179), graft use in the “no rhBMP-2” group was not further defined. One possibility is that the no rhBMP-2 group included patients who were implanted with iliac crest bone grafts (ICBGs), which have long been associated with higher index and follow-up costs versus rhBMP-2 [19, 20]. In the current study, the reason for such disparities in 24-month follow-up hospitalization charges between V-CBA and rhBMP-2 in the absence of differences in LOS remains unexplained and warrants further research.

Relatedly, an early cost-utility analysis [8] of 1- and 2-level dorsal lumbar fusions using ICBG with and without rhBMP-2 found that adding rhBMP-2 to ICBG was not cost-effective at 2 years in terms of cost-per-gain in quality-adjusted life years. However, a more recent cost-utility analysis [9] of patients undergoing spinal fusion at 5 or more levels reported that use of rhBMP-2 was associated with significant reductions in revisions for pseudarthrosis, which alone more-than doubled the 2-year direct costs to patients from an average of $61,000 to $138,000. Hence, the authors concluded that rhBMP-2 may yet be cost effective purely in terms of its ability to reduce the rate of pseudarthrosis, and other studies have supported this assertion [21, 22].

From this point of view, the present finding that use of V-CBA is associated with a significantly lower incidence of subsequent lumbar fusion procedures in the full cohort (10.12% vs 12.00% for rhBMP-2; Table 2) is of note, although this difference was not reflected in the single-level cohort, where the rates of subsequent lumbar fusion procedures were similar. While not a pure measure of pseudarthrosis, such cases would be represented in these numbers, which therefore do not fully explain the charge disparities presently observed. However, they do suggest that the clinical efficacy of V-CBA is at least equivalent to rhBMP-2 in terms of what is reported to be its most cost-effective attribute; namely, a lower incidence of pseudarthrosis.

Finally, as in the original study, the incidence of the majority of other potentially-relevant 24-month readmissions were similar between groups in both cohorts. Significantly higher rates of cardiac complications and pneumonia were observed presently in the V-CBA group versus the rhBMP-2 group (Table 2), which corresponded precisely to the significantly higher prevalence in related baseline comorbidities originally reported in the V-CBA group [10; Table 1], including cerebrovascular disease, congestive heart failure (single-level cohort only), chronic obstructive pulmonary disorder, diabetes with chronic complications, myocardial infarction, and peripheral vascular disease. Therefore, the present differences in 24-month follow-up readmissions between V-CBA and rhBMP-2 are expected and align with corresponding differences in initial comorbidities.

As with any study based on large healthcare databases, this study has inherent limitations. First, the data utilized here reflect the dollar amount that was charged for patient services, which may not reflect the final cost to the patient or third-party claims paid to the hospital or provider. Further, while this study utilized high-quality economic and clinical data, some potentially-relevant patient and procedure details were unavailable, such as extended medical histories, surgical approaches used, and fusion outcomes, which may have helped differentiate factors affecting charges and clinical outcomes. As well, some patients may have received follow-up treatment outside of the Premier Healthcare System, making such data unavailable. Finally, the present data represented economic and clinical information from US hospitals only and thus did not permit characterization for other regions.

## Conclusions

The results of this study suggest that the use of V-CBA for lumbar fusion surgeries performed in the US is associated with substantially lower 24-month follow-up hospitalization charges versus rhBMP-2, with both exhibiting similar rates of subsequent lumbar fusion procedures and potentially-relevant readmissions.

## Data Availability

The data that support the findings of this study are available from Premier Healthcare Solutions, Inc. (Charlotte NC). Restrictions apply to the availability of these data, which were used under license for the current study and so are not publicly available. However, data are available from the authors upon reasonable request and with permission of Premier Healthcare Solutions, Inc.

## Abbreviations

CBA: Cellular bone allograft
CCI: Charlson comorbidity index
CFR: US Code of Federal Regulations
CI: Confidence interval
ICBG: Iliac crest bone graft
LOS: Lengths of stay
PHD: Premier Healthcare Database
rhBMP-2: Infuse recombinant human bone morphogenetic protein-2 with a bovine collagen sponge scaffold
SD: Standard deviation
V-CBA: ViviGen cellular bone allograft
